# Enhancing Computational Thinking and Programming Logic Skills with App Inventor 2 and Robotics: Effects on Learning Outcomes, Motivation, and Cognitive Load

**DOI:** 10.3390/s25227059

**Published:** 2025-11-19

**Authors:** Yu-Ting Huang, Chien-Lung Li, Chin-Chih Chang, Wernhuar Tarng

**Affiliations:** 1 Institute of Learning Sciences and Technologies, National Tsing Hua University, Hsinchu 30013, Taiwan; joanne88012@gapp.nthu.edu.tw; 2 College of Computer Science and Electrical Engineering, Chung Hua University, Hsinchu 30012, Taiwan; bun520forever@gapp.nthu.edu.tw; 3 Department of Computer Science and Information Engineering, Chung Hua University, Hsinchu 30012, Taiwan; changc@chu.edu.tw

**Keywords:** robotic arms, App Inventor 2, computational thinking, programming logic, spatial reasoning, learning motivation, cognitive load, educational technology

## Abstract

**Highlights:**

**What are the main findings?**

- The instructional module integrating App Inventor 2 with a six-axis robotic arm significantly improved the students’ computational thinking and programming logic skills.
- Hands-on robotic operation and problem-based learning tasks enhanced the students’ intrinsic motivation, engagement, and willingness to tackle challenges.
- The task-based, visualized programming environment effectively reduced the students’ cognitive load by simplifying syntax and supporting concrete spatial reasoning.
- Students reported high system satisfaction, particularly regarding perceived usefulness, ease of use, and behavioral intention toward continued learning.

**What are the implications of the main findings?**

- Future research could develop and assess interdisciplinary learning scenarios to enhance students’ scientific literacy and learning outcomes.
- The proposed instructional module has demonstrated effectiveness in cultivating students’ cross-disciplinary integration skills within the context of this study’s specific school, age group, and short-term intervention.

**Abstract:**

Educational robotics (ER) has attracted growing attention as an effective means of cultivating computational thinking and programming skills through interactive, sensor-based learning environments. Integrating ER with visual programming platforms enables learners to engage in hands-on, technology-driven problem solving within authentic contexts. This study aimed to investigate the effects of a task-oriented instructional module, grounded in constructivist and experiential learning theories, that integrated App Inventor 2 with a six-axis robotic arm on junior high school students’ learning performance. A quasi-experimental design was conducted with 74 eighth-grade students from a junior high school in Hsinchu, Taiwan. The experimental group (*n* = 37) engaged in hands-on programming and robotic arm operations, whereas the control group (*n* = 37) received equivalent programming instruction with video demonstrations. Results indicated that the experimental group achieved significantly higher scores in spatial understanding, computational thinking, and programming logic. Students also reported greater motivation, lower cognitive load, and higher satisfaction with the integrated system. These findings suggest that combining App Inventor 2 with a physical robotic arm provides an effective framework for promoting computational thinking, motivation, and system interaction in technology education and smart learning environments.

## 1. Introduction

With the rapid advancement of artificial intelligence (AI) and automation technologies, robotic systems have been increasingly adopted across diverse fields such as industry, healthcare, services, and education. The integration of AI with advanced robotic mechanisms has given rise to AI-driven robots, whose capabilities and applications continue to expand rapidly. Among these, Educational Robotics (ER) has emerged as an effective approach to enhance students’ scientific literacy and computational thinking (CT). By combining programming logic with hands-on operation, ER enables learners to engage in constructive, trial-and-error learning processes that promote the understanding of abstract concepts, logical reasoning, and problem-solving skills [1,2]. These processes are closely aligned with constructivist learning theory, which emphasizes knowledge construction through active engagement, and experiential learning theory.

In recent years, many countries have actively promoted ER curricula and competitions—such as VEX Robotics and the World Robot Olympiad—to foster interdisciplinary learning and creativity among students [3]. While visual programming environments like App Inventor 2 simplify syntax learning, empirical evidence on their integration with multi-axis robotic arms to enhance computational thinking, programming logic, and engagement remains limited. This study addresses this gap by examining how a task-oriented module combining App Inventor 2 with a six-axis robotic arm impacts students’ learning outcomes, motivation, and cognitive load. These block-based tools simplify syntax learning, reduce cognitive load, and facilitate integration with Android sensors and IoT devices, making them highly suitable for designing interactive, task-oriented learning experiences in science and technology education.

Among the various ER applications, multi-axis robotic arms are particularly effective in cultivating key skills such as coordinate positioning, spatial reasoning, and process control—competencies closely aligned with the curricular goals of mathematics, physics, and technology education. Building upon this potential, the present study developed a task-oriented instructional module integrating App Inventor 2 with the WLKATA robotic arm, grounded in constructivist and experiential learning theories. The module guided students to program robotic arm movements and operational strategies using logical structures such as If–Else and For Loop. Learners engaged in an integrated perception–thinking–action process, reflecting principles of learning by doing and active knowledge construction in computational thinking and programming logic skills.

This study aimed to investigate the effects of the integrated instructional module on junior high school students’ computational thinking and programming logic, addressing the challenges of abstraction and low motivation often associated with traditional lecture-based instruction. The overarching goal is to enhance students’ conceptual understanding of technological applications while fostering sustained engagement and motivation toward computer programming and technology application.

To evaluate the effectiveness of this module, a quasi-experimental design was employed. The experimental group engaged in interactive, hands-on learning using the robotic arm integrated with App Inventor 2, whereas the control group received equivalent instruction via App Inventor 2 supported by video demonstrations. Both groups completed achievement tests and surveys assessing learning performance, motivation, and cognitive load, with the experimental group additionally completing a system satisfaction survey. Furthermore, semi-structured interviews were conducted to capture the students’ learning experiences and feedback, providing a qualitative complement to the quantitative results. These interviews served to obtain richer insights into the students’ perceptions and engagement that might not be fully reflected in quantitative data.

The objective of this study was to develop and evaluate a task-oriented instructional module integrating App Inventor 2 with a six-axis robotic arm, grounded in constructivist and experiential learning theories, to enhance junior high school students’ computational thinking, programming logic skills, learning motivation, and cognitive load management through hands-on, interactive, and technology-supported learning experiences. The research questions are as follows:

(1) Does the teaching module integrating App Inventor 2 with interactive robotic-arm operation improve students’ computational thinking and programming logic skills?
(2) How does hands-on interaction with the robotic arm affect intrinsic and extrinsic learning motivation, compared with video-based instruction?
(3) Does the module reduce cognitive load, as measured by mental load and mental effort scales, compared with video-based instruction?
(4) How do students evaluate system satisfaction, particularly regarding perceived usefulness, perceived ease of use, and behavioral intention of the integrated App Inventor 2 and robotic arm system?

## 2. Literature Review

### 2.1. Robotics and STEM Education

**Figure 1:**
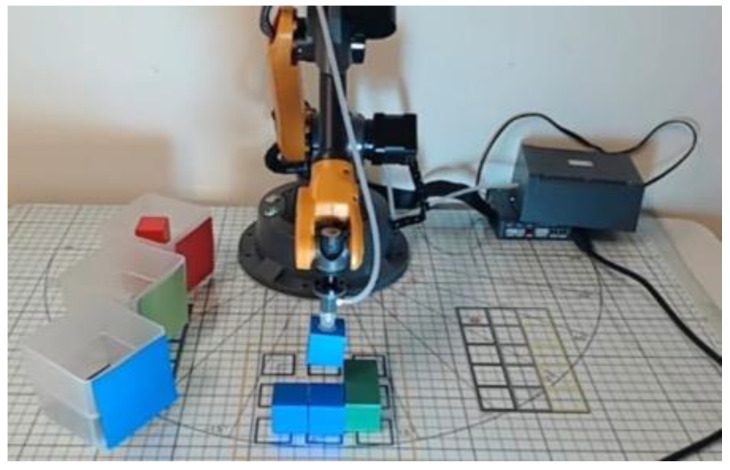
Application of the WLKATA Mirobot in STEM education.

Robots are intelligent mechanical systems capable of sensing, actuation, and autonomous control, applied across fields such as manufacturing, healthcare, agriculture, and education [4]. Educational robotics (ER) integrates related technologies into learning environments, allowing students to cultivate computational thinking (CT), programming logic, and problem-solving skills within STEM disciplines [5,6]. Among the ER applications, multi-axis robotic arms have gained popularity in secondary education (Figure 1) due to their programmable control and multi-degree-of-freedom motion, allowing students to simulate authentic industrial processes [7]. Evidence suggests that robotic-arm activities improve spatial reasoning, programming skills, engagement, and confidence in problem-solving [8]. However, existing research primarily focuses on university or high school contexts and software-only programming tasks, leaving a gap regarding junior high students’ cognitive and motivational outcomes when combining block-based programming with physical robotic arms.

Although visual programming environments like App Inventor 2 and robotic arms have been studied individually, little research has examined their integration in task-oriented modules. Prior studies have focused on technical skills or isolated learning outcomes, neglecting how combined, hands-on experiences influence computational thinking, programming logic, cognitive load, and motivation. Furthermore, the iterative problem-solving processes inherent in multi-axis robotic arm programming remain underexplored, limiting our understanding of how integrated, technology-supported learning fosters both conceptual understanding and sustained engagement in secondary education. The present study addresses this gap by integrating task-oriented robotic-arm activities with App Inventor 2 to foster experiential learning through perception–action–reflection cycles. This integration is expected to concretize abstract programming and spatial concepts, aligning learning with real-world applications.

### 2.2. App Inventor 2

**Figure 2:**
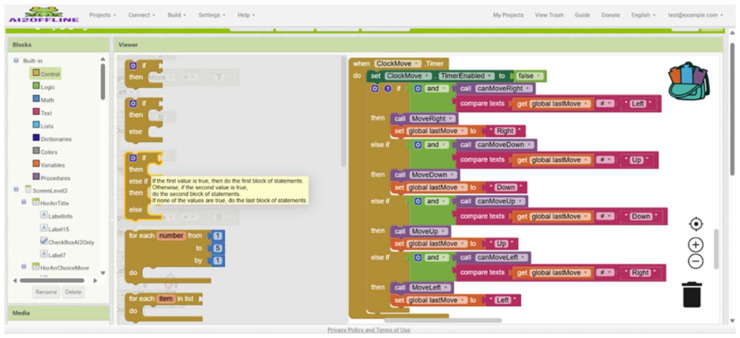
User interface of the App Inventor 2 programming environment.

App Inventor 2, developed by Google and now maintained by MIT, is a block-based visual programming platform [9] that lowers entry barriers for novices by allowing drag-and-drop coding for Android apps (Figure 2). Unlike Scratch, it emphasizes mobile app development and sensor/IoT integration, enabling students to connect abstract computational concepts with real-world tasks [10]. While prior studies have reported that App Inventor 2 can improve computational thinking and problem-solving skills [11,12,13,14,15,16], findings are often descriptive and context-specific, focusing on task completion rather than measuring cognitive strategies, algorithmic reasoning, or logical structure application.

Few studies have explored its combination with physical robotics, which may enhance spatial reasoning and motivation through embodied interaction, a critical yet under-investigated mechanism. By integrating App Inventor 2 with a six-axis robotic arm, this study examines how visual programming tools paired with tangible, hands-on experiences can enhance both CT and programming logic skills in junior high learners.

### 2.3. Computational Thinking

Computational thinking (CT) is a problem-solving framework encompassing decomposition, pattern recognition, abstraction, and algorithmic design, promoting logical reasoning, systematic problem-solving, and digital literacy [1,5,17,18,19]. While traditional text-based programming can be abstract and cognitively demanding for beginners, visual programming platforms such as App Inventor 2 provide concrete, manipulable representations of logic structures, facilitating learning, engagement, and motivation [20].

Prior studies suggest that App Inventor 2 can enhance CT, but most evidence is limited to software-only tasks or single CT dimensions, often measured indirectly through task completion, code correctness, or teacher ratings. These approaches rarely capture higher-order reasoning, integrated problem-solving, or spatial cognition, particularly in younger learners. Game-based and interactive programming interventions have shown additional benefits, improving CT, engagement, and motivation across K–12 contexts.

Most prior studies measured computational thinking using task-based assessments, coding project performance, or structured pre/post-tests evaluating decomposition, algorithmic reasoning, and problem-solving accuracy [21,22,23,24]. Gamification and educational robotics studies often assessed CT through performance metrics (e.g., correct program sequences, task completion) and process measures (e.g., strategy use, iterative problem-solving). These approaches demonstrate that interactive, hands-on, and game-like tasks can enhance both logical reasoning and engagement. This evidence matters to our intervention because integrating App Inventor 2 with a six-axis robotic arm similarly combines task-oriented programming with embodied spatial interaction, targeting multi-dimensional CT development beyond software-only contexts.

Most prior studies on App Inventor 2 assessed CT through software-only tasks, task completion, code correctness, or teacher ratings, often neglecting higher-order reasoning, integrated problem-solving, and spatial cognition, particularly among younger learners. In contrast, this study integrated App Inventor 2 with a six-axis robotic arm, providing embodied, task-oriented experiences that link programming logic to spatial and operational contexts. This approach enables the simultaneous development of programming logic, spatial reasoning, and multi-dimensional CT, addressing gaps in prior research and fostering authentic, interactive learning.

In this study, hands-on interaction with robotic arms provides embodied experiences that link programming logic to spatial and operational contexts. Embedding CT tasks in a task-oriented, physical module allows students to simultaneously develop programming logic, spatial reasoning, and problem-solving strategies. This approach addresses gaps in prior research, particularly for junior high students, and enables the assessment of multi-dimensional CT outcomes in authentic, interactive contexts.

### 2.4. Spatial Concepts

Spatial ability—the capacity to perceive, manipulate, and interpret visual-spatial information—is foundational in STEM learning [25,26,27]. Core components include:

- Mental rotation—imagining objects in different orientations; developed via VR, 3D simulations, and robotic manipulation [28,29,30].
- Spatial orientation—understanding relative positions; strengthened by visual cues and task scaffolding.
- Coordinate localization—mapping x–y positions to spatial contexts; reinforced through hands-on manipulation of robotic arms.

Research demonstrates that robotic interactions concretize abstract spatial concepts, enhancing visualization, geometric reasoning, and task accuracy [31,32,33,34]. Integrating spatial tasks and robotic arm operations with App Inventor 2 allows learners to link programming logic with physical movement, strengthening mental models of coordinate systems and supporting interdisciplinary STEM reasoning.

However, many prior studies relied on simple performance metrics or task completion, which may not validly capture multi-dimensional spatial reasoning. They often overlook integrated skills such as mental rotation, spatial orientation, and coordinate localization within authentic problem-solving contexts. This study addresses these limitations by embedding spatial assessments within task-oriented, hands-on robotic interactions, enabling learners to link programming logic with physical movement. This approach offers a more valid, comprehensive evaluation of spatial ability while fostering interdisciplinary STEM reasoning and applied learning outcomes.

### 2.5. Learning Motivation

Motivation drives engagement, persistence, and learning outcomes [35,36]. Self-Determination Theory (SDT) distinguishes intrinsic motivation (interest, challenge enjoyment) from extrinsic motivation (rewards, evaluations) [37,38]. While technology adoption studies often reported increased motivation, many focused on system use rather than task engagement or learning outcomes. Evidence indicates that interactive, hands-on robotics enhances intrinsic motivation, interest, and challenge acceptance [39,40]. By combining App Inventor 2 with robotic-arm operation, the present study leverages active, goal-directed learning to sustain engagement and transform extrinsic cues (e.g., task completion) into internalized, self-driven learning motivation.

Prior studies assessed learning motivation using self-report questionnaires or system usage data, which may not validly capture task-specific engagement or sustained intrinsic motivation. This study extended previous research by embedding motivational assessments within authentic, interactive robotic tasks that integrate App Inventor programming with physical operation, enabling a more precise evaluation of both intrinsic and extrinsic motivational processes and offering deeper insight into how interactive, task-oriented learning fosters self-driven, goal-directed engagement.

### 2.6. Cognitive Load

**Figure 3:**
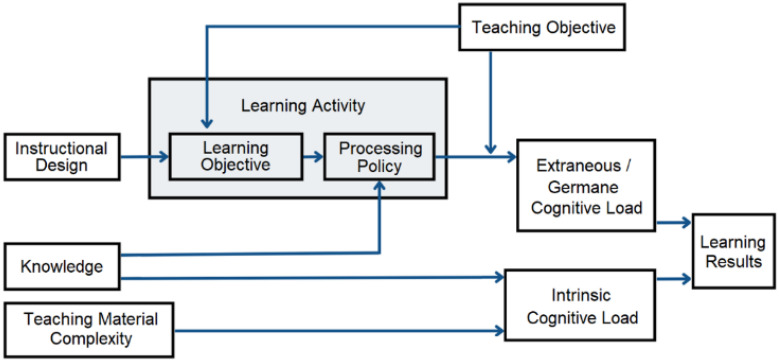
Theoretical framework illustrating the three types of cognitive load.

Cognitive Load Theory (CLT) emphasizes the limits of working memory and the need to balance intrinsic, extraneous, and germane load for effective learning [41]. Poor instructional design or abstract learning materials increase extraneous load, hindering CT and programming acquisition. According to Sweller [42], cognitive load consists of three types (Figure 3): intrinsic load, which arises from the complexity of materials and the learner’s prior knowledge; extraneous load, caused by ineffective instructional design or presentation that diverts cognitive resources; and germane load, referring to the mental effort invested in understanding and constructing knowledge. To differentiate task difficulty from mental effort, Paas further distinguished between mental load and mental effort, providing a framework for assessing cognitive load [43].

While prior studies commonly measured cognitive load through subjective ratings or task performance, they often failed to distinguish mental effort from task difficulty or capture real-time fluctuations during complex, hands-on activities. By embedding assessments within interactive, task-oriented robotic programming, this study provides a more valid, refined evaluation of intrinsic, extraneous, and germane load, linking cognitive processes directly to learning outcomes in computational thinking and programming.

Building on the above foundation, this study employed subjective rating scales to measure cognitive load during the intervention, offering insights into the instructional design’s effectiveness in managing cognitive resources and informing future curriculum and activity improvements. Hands-on robotic tasks can reduce extraneous load by concretizing abstract concepts and increase germane load by promoting active problem-solving and reflection. However, few studies have empirically measured cognitive load in junior high learners engaging with both programming and robotics simultaneously, a gap this study addressed using subjective rating scales aligned with CLT principles.

### 2.7. Technology Acceptance Model

**Figure 4:**
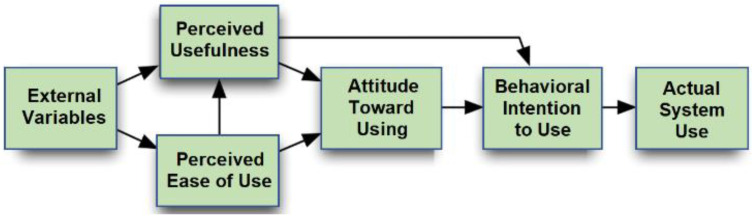
Technology Acceptance Model II (TAM2) showing key determinants.

The Technology Acceptance Model (TAM) [44] was developed based on the Theory of Reasoned Action [45] and provides a framework for understanding users’ perceptions and behavioral intentions toward adopting new technologies. TAM posits that Perceived Usefulness (PU) and Perceived Ease of Use (PEOU) are the primary determinants of users’ attitudes and intentions, which in turn influence the actual system usage [46]. To enhance its explanatory and predictive power, TAM was extended to TAM II (Figure 4) by incorporating external variables [47].

The Technology Acceptance Model (TAM) predicts system adoption based on PU and PEOU [48,49,50]. While TAM is often used to assess general acceptance, its direct link to academic engagement is limited. In this study, TAM was employed to evaluate the students’ perceptions of the App Inventor 2 and robotic arm system. High acceptance is expected to facilitate hands-on engagement in task-oriented programming activities, allowing students to focus cognitive resources on learning computational thinking and programming logic skills, while motivation and engagement are conceptualized through SDT. This combination ensures that technical usability complements, rather than substitutes, the mechanisms that drive learning outcomes.

### 2.8. Constructivist and Experiential Learning Theories

Constructivist and experiential learning theories provide the theoretical foundation for integrating robotic arms in programming education. Constructivism posits that learners actively construct knowledge through interaction with the environment and reflection on their experiences [51]. In the context of educational robotics, this approach enables students to build understanding by designing, programming, and testing robotic actions, thereby linking abstract concepts to tangible outcomes. Similarly, experiential learning theory emphasizes the cyclical process of concrete experience, reflective observation, abstract conceptualization, and active experimentation [52].

When students engage in the hands-on programming of physical robotic arms, they undergo these learning cycles, deepening their cognitive engagement and problem-solving abilities. Prior studies [53] have shown that robotic-based experiential learning enhances learners’ computational thinking, motivation, and conceptual understanding. These frameworks together support the design of this study’s task-oriented module, aligning learning-by-doing activities with constructivist and experiential principles to foster meaningful, interdisciplinary learning outcomes.

## 3. Materials and Methods

### 3.1. Research Design and Level

**Figure 5:**
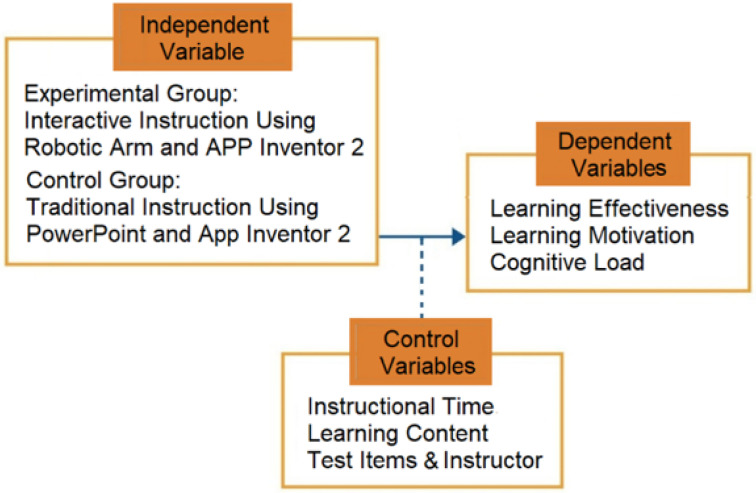
Research variables in the quasi-experimental design.

This study employed a quasi-experimental design at the class level, representing a group comparison experimental study within the research pyramid (intervention-based educational research). The study involved 74 eighth-grade students from a junior high school in Hsinchu, Taiwan, with 37 assigned to the experimental group and 37 to the control group. The experimental group engaged in hands-on activities using a physical robotic arm programmed via App Inventor 2, whereas the control group received equivalent instruction in computational thinking and programming logic with App Inventor 2, supported by video demonstrations of robotic operations. Research variables in the quasi-experimental design are shown in Figure 5.

- Independent variable: Instructional activity (hands-on vs. video-based instruction);
- Dependent variables: Learning achievement, learning motivation, cognitive load, and system satisfaction;
- Control variables: The intervention lasted three weeks, with one 50 min session per week for a total 150 of minutes. To enhance internal validity, the instructional time, content, assessment items, and instructor were kept consistent, and pretest scores were used as covariates in the ANCOVA to control for initial differences. Participants were selected to have no prior experience with the target learning content.

### 3.2. Research Instruments

To evaluate research objectives and questions, four quantitative instruments were used, along with qualitative interviews to provide complementary insights.

#### 3.2.1. Learning Achievement Test

A self-developed achievement test assessed knowledge in three domains—robotics and spatial coordinates, computational thinking, and programming logic—covering three cognitive levels (understanding, applying, analyzing) based on the revised Bloom’s taxonomy [54]. Test items were refined through expert review and pilot testing, resulting in 15 multiple-choice questions (Appendix A), including selected tasks from the Bebras International Computational Thinking Challenge [55] for content validity (Table 1).

#### 3.2.2. Learning Motivation Scale

The learning motivation scale employed in this study was adapted from the instrument originally developed by Pintrich [56] and later revised by Wang and Chen [57]. The final version was refined through expert review and consultation with domain specialists to ensure that its content was appropriate, accurate, and valid. The scale consisted of six items across two dimensions—intrinsic motivation and extrinsic motivation—with three items assigned to each subscale individually.

#### 3.2.3. Cognitive Load Scale

The cognitive load scale, based on Paas’s [43] theoretical framework, was designed to assess the learners’ cognitive burden during instruction. It includes eight items—five evaluating mental load and three assessing mental effort—providing a reliable measure of cognitive load in learning contexts.

#### 3.2.4. System Satisfaction Questionnaire

To evaluate the students’ acceptance and attitudes toward the instructional module, the experimental group completed a system satisfaction survey following the intervention. Based on Davis’ Technology Acceptance Model [46] and refined through expert review, the questionnaire includes nine items across three dimensions: Perceived Usefulness (3 items), Perceived Ease of Use (3 items), and Behavioral Intention (3 items).

### 3.3. Experimental Design

Constrained by time, sample size, and teaching resources, this study employed a quasi-experimental design to examine the effects of an instructional model on junior high school students’ learning outcomes. The experimental group engaged in hands-on activities with App Inventor 2 programming and robotic-arm operation to develop computational thinking and programming logic skills, while the control group received equivalent programming instruction supported by video demonstrations (Table 2).

The coding for the experimental procedure is listed as follows:

- O1: Both groups completed a pre-test on programming logic and computational thinking.
- X1: The experimental group received instruction with robotic-arm operations.
- X2: The control group received instruction supported by video demonstrations.
- O2: Both groups completed a post-test on programming logic and computational thinking.
- O3: Both groups completed questionnaires measuring learning motivation and cognitive load.
- O4: The experimental group completed an additional TAM questionnaire.

The study was conducted with two eighth-grade classes in a junior high school over three sessions for a total of 150 min. The classes were randomly assigned to the experimental group and the control group, which helped reduce selection bias. However, because randomization occurred at the class level rather than the individual level, potential threats such as pre-existing group differences could not be ruled out. To mitigate these effects, the instructional content, duration, and instructor were kept consistent, and pretest scores were used as covariates in the ANCOVA analysis to control for initial differences, thereby enhancing the internal validity of the comparisons.

**Figure 6:**
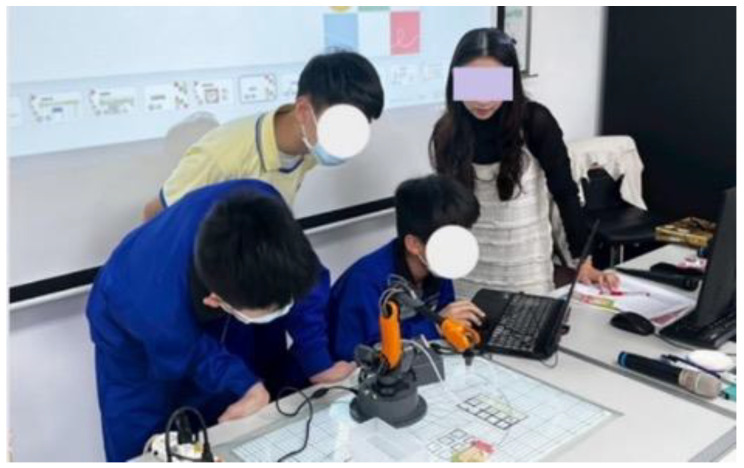
Experimental group learning with a physical robotic arm.

Prior to the instructional intervention, both the experimental and control groups completed a pre-test to assess baseline programming knowledge and problem-solving skills. This ensured that any observed learning gains could be attributed to instructional activities rather than prior knowledge differences. Subsequently, the experimental group engaged in hands-on programming activities using a physical robotic arm (Figure 6). The core activity, “Smart Maze”, required students to:

- Program the robotic arm using App Inventor 2 to navigate a predefined maze.
- Translate logical commands into visual programming blocks within App Inventor 2.
- Test and refine their programs to achieve the correct maze navigation.

The hands-on tasks were designed to enhance problem-solving and computational thinking skills. All activities were standardized, and task instructions, robot setup, and maze layouts were pilot-tested with a small group of students to ensure clarity, feasibility, and appropriate difficulty before the formal experiment.

**Figure 7:**
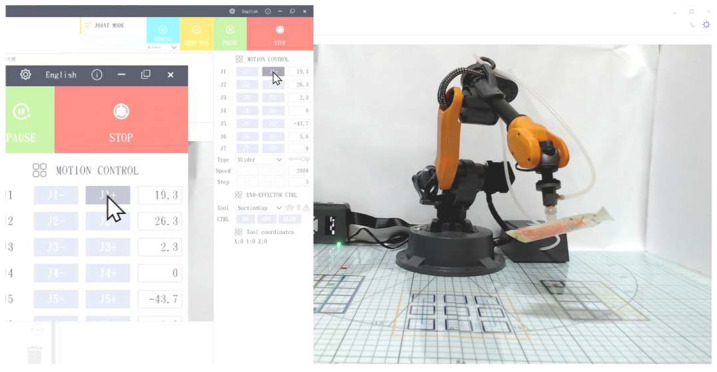
Instructional video demonstrating robotic-arm operation for the control group.

The control group received equivalent instruction in App Inventor 2 programming but through video demonstrations of the robotic-arm operation (Figure 7):

- Step-by-step explanations of robotic-arm programming using App Inventor 2.
- Visual demonstrations of the Smart Maze task being completed by the robotic arm.
- Completing equivalent task challenges presented in the experimental group using App Inventor 2, ensuring that both groups were exposed to the same content.

**Figure 8:**
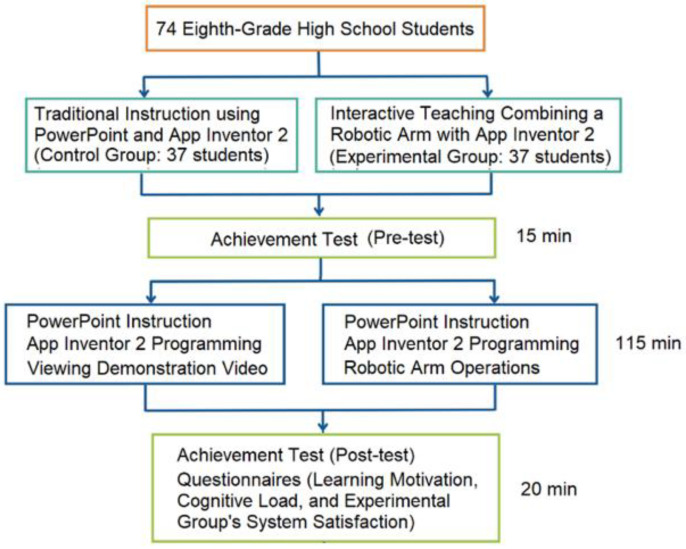
Research process for the two groups.

This experimental design—incorporating pilot-tested tasks, validated instructional materials, and consistent instructional conditions in terms of time allocation, learning content, and task standardization—was intended to ensure reproducibility, reliability, and fairness in evaluating the effects of hands-on versus video-based instruction. Following the intervention, both groups completed a post-test to assess learning effectiveness. Furthermore, they also filled out scales measuring learning motivation and cognitive load, with the experimental group additionally completing a system satisfaction survey. The research process for the two groups is shown in Figure 8.

This study was conducted in a computer classroom with participants drawn from two eighth-grade classes, where instructional activities were implemented as a group-level intervention. A quantitative approach was employed using a self-developed learning achievement test, a motivation scale, a cognitive load scale, and a system satisfaction questionnaire to measure the students’ knowledge, motivation, and cognitive engagement. Complementing this, a qualitative approach included semi-structured interviews with selected students to capture their learning experiences and perceptions. This intervention study spanned three weeks, with one 50 min session per week, allowing the pre-test, treatment, and post-t assessments to assess changes over time.

### 3.4. Instructional Design

In this study, the instructional module was developed using a competency-based approach, aligned with the Curriculum Guidelines of 12-Year Basic Education issued by the Ministry of Education (MOE), Taiwan [17]. To incorporate the educational values of initiative, interaction, and collective learning, the module integrates the App Inventor 2 visual programming platform with the WLKATA six-axis robotic arm, and features hands-on interdisciplinary learning activities. The instructional goals focus on developing students’ scientific literacy, programming logic, computational thinking, and problem-solving skills, emphasizing the principle of learning by doing. It can also address core competencies such as A2: Systems Thinking and A3: Innovation and Adaptability in the Curriculum Guidelines of 12-Year Basic Education.

#### 3.4.1. Course Content

The course lasted three weeks, with one 50 min session per week, and focused on an interactive task, “Smart Maze,” integrating App Inventor 2 with a six-axis robotic arm. Four extension activities were also incorporated to develop the students’ programming logic, spatial reasoning, computational thinking, and problem-solving skills. The first week focused on introducing programming logic and spatial concepts, including basic operation of App Inventor 2 and the robotic arm interface. After that, the experimental group engaged in hands-on operation to explore human–machine interaction, while the control group received introductory programming instruction using App Inventor 2, supported by video demonstrations to enhance conceptual understanding.

In the second week, the instruction emphasized programming logic structures, specifically conditional statements (If–Else) and loops (For Loop). Students applied these concepts to perform the Smart Maze task, observing how programming logic governs the robotic arm’s operation in three-dimensional space. This approach facilitated the internalization of abstract concepts and strengthened the integration of spatial orientation with computational thinking. The experimental group actively developed and executed block-based programs, directly observing the robotic arm’s responses to reinforce understanding, whereas the control group received equivalent instruction in programming logic, supported by video demonstrations and teacher explanations.

**Figure 9:**
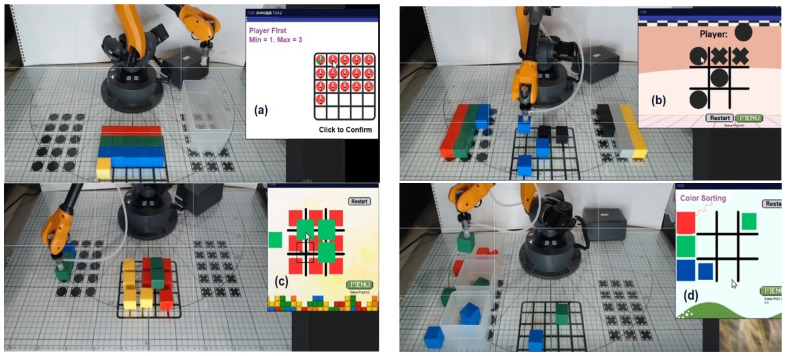
(**a**) Removing Blocks, (**b**) Tic-Tac-Toe, (**c**) Block Stacking, and (**d**) Color Sorting.

In the third week, both groups focused on extension and practical applications (Figure 9), completing four hands-on tasks: “Removing Blocks”, “Tic-Tac-Toe”, “Block Stacking”, and “Color Sorting”, as described below:

- Removing Blocks: A two-player Nim game in which players take turns removing blocks from distinct heaps or piles. On each turn, a player must remove at least one block and may take any number from a single heap, as defined at the start. The player forced to take the final block loses the game.
- Tic-Tac-Toe: A strategy game played on a 3 × 3 grid. Players take turns placing their symbol—either “X” or “O”—in an empty square. The objective is to align three of one’s symbols in a row—horizontally, vertically, or diagonally—before the opponent. If all squares are filled without a winning line, the game ends in a draw.
- Block Stacking: This hands-on activity requires players to use a programmed robotic arm to pick up and stack blocks to form specified shapes according to instructions. The task demands precision, careful planning, and spatial reasoning, as the robot must execute each command accurately.
- Color Sorting: In this task, students program a robotic arm to identify and sort blocks by color using a robotic vision system. The activity requires designing logical rules to ensure accurate classification of the blocks.

The instructional activities required students to actively manipulate a robotic arm, providing hands-on experience that reinforced learning and fostered their programming logic, computational thinking, and overall technological literacy. To develop an integrated programming environment, this study combined App Inventor 2 with a physical robotic arm as the instructional module. An interactive operation platform was created to provide students with practical learning experience, allowing them to directly manipulate the robotic arm while learning programming logic, spatial reasoning, and coordinate concepts. This approach bridged abstract concepts in programming logic with hands-on operations, making learning more concrete and intuitive.

The system design comprised three core components: (1) the physical robotic arm, which executed student-generated commands and provided real-time visual feedback; (2) the App Inventor 2 user interface, which enabled students to construct logic flows using a block-based, drag-and-drop programming environment; and (3) an intermediary control module to facilitate communication between the programming interface and the robotic arm, ensuring smooth execution of user commands and integration of abstract programming concepts with physical manipulation of the robotic arm.

#### 3.4.2. WLKATA Mirobot

This study utilized the WLKATA Mirobot as the learning tool, a six-axis robotic arm known for its flexible joint structure and precise motion control, commonly used in education and smart manufacturing simulation. The robotic arm performs movement and rotation tasks in three-dimensional space (X, Y, Z), helping learners understand mechanical motion principles and spatial coordinate system concepts. By programming commands to control the robot’s actions, students receive real-time visual feedback, reinforcing the link between programming logic and abstract concepts while supporting the development of higher-order thinking and problem-solving skills.

Using forward kinematics, the six-axis robotic arm calculates the precise position and orientation of its end effector based on the specified joint angles and link parameters. Each joint contributes rotational or translational movement, mathematically represented by the transformation matrices derived from Denavit–Hartenberg (DH) conventions [30]. By sequentially multiplying these matrices from the base joint to the end effector, learners can determine the cumulative displacement and orientation of each joint in Cartesian space. This process provides precise spatial information, allowing the robotic arm to validate motion planning and execute tasks accurately. A robotic arm uses inverse kinematics to convert a target position into the joint angles needed for the end effector to reach it accurately. For object detection, the robotic vision system captures an image, isolates the object, and identifies key features—such as color, shape, and position—which are converted into spatial coordinates and object types to guide precise manipulation.

#### 3.4.3. App Inventor 2 Programming

**Figure 10:**
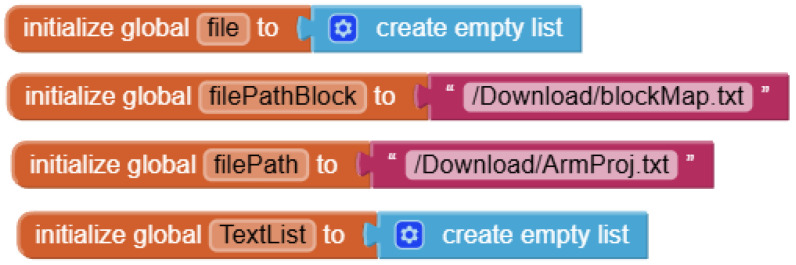
Setting path for reading text files in App Inventor 2.

App Inventor 2 is a block-based visual programming platform designed for easy mobile application development, making it particularly suitable for beginners. In this study, students used App Inventor 2 to design a control interface through the COM port with features such as directional control, task selection, and coordinate setting to operate the robotic arm. To enable communication between an app and a COM port, the students’ commands were saved as a local text file, which was then retrieved and parsed using the file-reading function of App Inventor 2 (Figure 10), allowing the intermediary control module to execute corresponding tasks through the robotic arm.

**Figure 11:**
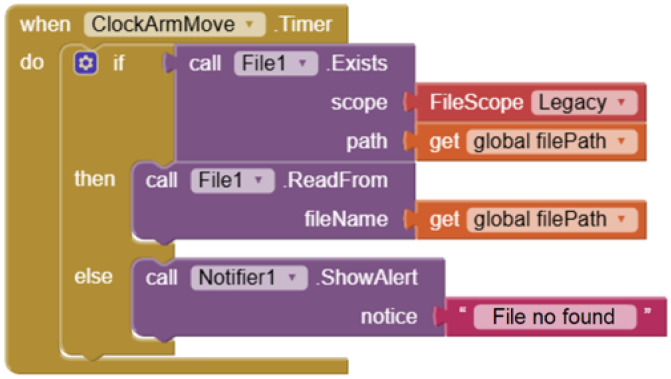
Reading a text file using the Clock component in App Inventor 2.

In addition, the user interface of App Inventor 2 includes a built-in checking mechanism, which utilizes a timer to continuously read status flags (e.g., Status:Proj2:Arm) to verify whether the task command has been completed. This feature ensures the continuity and accuracy of the operation process (Figure 11).

#### 3.4.4. Communication Interface

**Figure 12:**
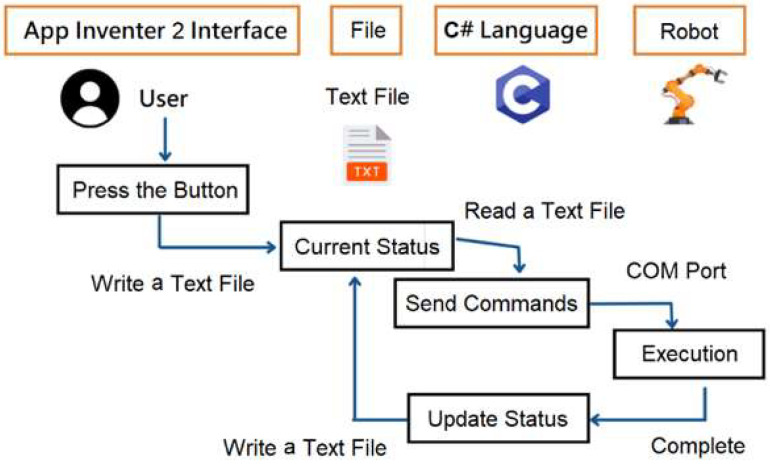
Integrating App Inventor 2 with the WLKATA robotic arm.

To enable communication between App Inventor 2 and the robotic arm, an intermediary control module was developed using C# programming language. The module translates app-generated commands into instructions that the robotic arm can execute. Communication with the robotic arm is established via a serial COM port. The operational procedure is as follows: when students interact with the user program, the generated commands are saved to text files. The C# control module reads these files, converts them into executable commands, and transmits the commands to the robotic arm. Upon task completion, the robotic arm writes the results back to the text file, enabling the user program to monitor task status and form a closed-loop control system (Figure 12).

The C# control module also includes functions such as axis adjustment, coordinate calibration, and task execution, allowing the teacher to support task initialization and manage system operations during instruction. Although the control interface was operated exclusively by the teacher, it played a crucial role in ensuring task readiness, execution accuracy, smooth system operation, and effective classroom management. By integrating hardware and software, students developed programming logic, abstract thinking, and problem-solving skills through hands-on practice. This approach also enhanced their technological literacy and interdisciplinary integration abilities.

### 3.5. Data Analysis

In this study, data were collected from pre- and post-test scores, responses to the learning motivation and cognitive load scales, and the system satisfaction questionnaire, which was completed exclusively by the experimental group. In addition, semi-structured interviews were conducted to capture the students’ learning experiences and feedback, providing a qualitative complement to quantitative results. The data analysis procedures and statistical methods adopted are summarized as follows.

#### 3.5.1. Source of Information

The experimental results consisted of three components: (1) test scores from a 15-item learning achievement test assessing robotics and spatial coordinates, computational thinking, and programming logic; (2) survey data from a six-item learning motivation scale and an eight-item cognitive load scale administered to both groups, with the experimental group also completing a nine-item system satisfaction questionnaire; and (3) qualitative data from semi-structured interviews with five representative students from each group, capturing their perspectives on motivation and learning experiences and providing feedback to support the interpretation of questionnaire results.

#### 3.5.2. Data Processing

After collecting the experimental results, all test scores and questionnaire data were compiled and coded by the researchers. The data were first prepared in Microsoft Excel and then imported into IBM SPSS Statistics (Version 30) for analysis, with missing or extreme values removed to ensure integrity. The interview transcripts were analyzed thematically, with responses repeatedly compared against coded categories to ensure the reliability and validity of the qualitative findings. Cronbach’s alpha coefficients were calculated to assess the internal consistency and reliability of each questionnaire dimension:

- Learning Motivation: Overall = 0.854; Intrinsic Motivation = 0.823; Extrinsic Motivation = 0.715.
- Cognitive Load: Overall = 0.842; Mental Load = 0.771; Mental Effort = 0.750.
- System Satisfaction: Overall = 0.775; Perceived Usefulness = 0.708; Perceived Ease of Use = 0.730; Behavioral Intention = 0.850.

In addition to reliability testing, expert validity was conducted. Three domain experts in learning technology and computer education reviewed all instruments for content relevance, clarity, and alignment with research constructs. Based on their feedback, wording and item structure were refined to enhance content validity and ensure that each dimension accurately represented the intended theoretical constructs.

#### 3.5.3. Statistical Analysis Methods

In this study, four statistical methods were employed to evaluate the effects of the instructional intervention and test the research hypotheses: (1) descriptive statistics to summarize the means and standard deviations of pre-test, post-test, and questionnaire scores; (2) paired-samples *t*-tests to compare pre- and post-intervention differences within each group for learning achievement; (3) a two-way mixed-design analysis of variance (ANOVA) was conducted to examine the main and interaction effects of group and test, with simple main effects analyses conducted when significant interactions were detected; and (4) independent-samples *t*-tests to compare post-intervention scores between groups on intrinsic and extrinsic motivation, mental load, and mental effort, assessing the impact of instructional activities on psychological variables.

The reproducibility box for this study is described below:

- Software and versions: Data analyses conducted in SPSS 30.0 and MIT App Inventor 2 nb202b.
- Data cleaning rules: Participants with >20% missing responses or extreme outliers (>3 SD) were excluded, and missing values were handled by listwise deletion.
- Planned analyses: Pre–test and post-test comparisons with paired-samples *t*-tests; between-group differences with independent *t*-tests; overall effects analyzed with mixed-design ANOVA. Effect sizes reported with 95% CIs.
- Instrument: Achievement test is included in Appendix A.

#### 3.5.4. Qualitative Data Design, Treatment, and Analysis

To complement quantitative findings, qualitative data were collected through semi-structured interviews with five representative students from each group. The interview protocol focused on the students’ experiences, perceptions of instructional activities, motivation, problem-solving strategies, and challenges encountered during the Smart Maze and extension tasks. Interviews were conducted individually in a quiet classroom setting, audio-recorded with consent, and transcribed verbatim for analysis.

Data were analyzed thematically following Braun and Clarke’s approach [58]: initial coding identified meaningful units, which were then grouped into categories reflecting patterns in learning experiences, engagement, and problem-solving strategies. Codes and themes were iteratively refined through discussion among researchers to ensure reliability. Qualitative results were used to interpret and contextualize survey responses, providing insights into the students’ learning perceptions versus video-based instruction. Triangulation with questionnaire and test data strengthened the validity of the findings.

## 4. Analysis Results

This study conducted a teaching experiment with 74 eighth-grade students from a junior high school in Hsinchu, Taiwan to examine the effects of different instructional activities on learning outcomes, motivation, and cognitive load. Pre- and post-tests were used to measure learning achievement, motivation, and cognitive load, while the experimental group additionally completed a system satisfaction questionnaire to evaluate the acceptance of instructional design and learning experience. Furthermore, qualitative data were collected through semi-structured interviews to understand the students’ challenges, motivational shifts, and feedback toward the instructional module. The teaching experiment was approved by the institutional ethics committee. As the participants were minors, written informed consent was obtained from their parents or legal guardians, and assent was obtained from the students prior to participation. All data were anonymized, securely stored, and used solely for research purposes.

Thematic analysis was employed to identify qualitative insights that complemented the quantitative results by revealing the learners’ perspectives, learning experiences, and individual differences in understanding and engagement. The findings are outlined as follows: (1) comparisons of pre- and post-test scores across groups for learning outcomes; (2) analysis of the effects of instructional activities on motivation and cognitive load; (3) system satisfaction data from the experimental group; and (4) qualitative insights from interview data to enrich, interpret, and contextualize overall results.

### 4.1. Learning Effectiveness Analysis

To examine the impact of instructional activities on the students’ learning effectiveness, an achievement test with 15 items, covering computational thinking, programming logic skills, and spatial concepts was administered before and after the intervention. Each dimension was weighted equally (5 points). The results in Table 3 revealed positive effects for both instructional activities. Significantly, the experimental group demonstrated a greater score gain and a reduced score variance, suggesting improved effectiveness and decreased learning disparities. Overall, the integration of a physical robotic arm with interactive hands-on activities appears to enhance learning outcomes and support more equitable performance, showing promise for educational applications.

The Shapiro–Wilk test indicated that the pre-test scores for both groups did not significantly deviate from normality. Levene’s test confirmed homogeneity of variances, suggesting no significant differences in pre-test performance and supporting baseline equivalence. To compare learning gains before and after the instructional intervention, paired-sample *t*-tests were conducted for both groups. Results in Table 4 show that both groups improved, with the experimental group demonstrating a markedly greater gain, indicating that the proposed hands-on, visual programming approach was more effective in enhancing the students’ computational thinking and programming logic skills.

In summary, the results show that the experimental group outperformed the control group across all cognitive levels. For the separate levels, significant gains were observed in Understanding, Applying, and Analyzing, whereas the control group only showed a modest improvement in Understanding and Applying, with no change in Analyzing. The largest difference was in Applying, indicating that the task-oriented robotic arm module effectively strengthened the students’ higher-order cognitive skills and practical problem-solving abilities. Overall, these findings suggest that learning activities with robotic operation promotes deeper learning compared to video-based instruction.

Before conducting subsequent analyses, the equivalence of pre-test scores between the two groups was assessed to ensure a valid baseline. Levene’s test for equality of variances (*F* = 1.571, *p* = 0.214) and an independent-samples *t*-test (*t* = 1.684, *p* = 0.097) confirmed that there were no significant differences in pre-test performance, indicating that the assumption of baseline equivalence was met. Accordingly, a two-way mixed-design ANOVA was conducted to examine the effects of instructional activities and test phase (pre-test vs. post-test) on learning outcomes.

A significant main effect of the achievement test was found, indicating that the students’ learning performance improved significantly after the intervention (Table 5). Also, a significant main effect of instructional group was observed, suggesting that different teaching methods had a substantial impact on the students’ learning outcomes. The interaction between test and group was significant, indicating that the trends of improvement in learning outcomes differed between the two groups. Specifically, the experimental group demonstrated a significant improvement, with a greater gain compared to the control group. The effect size was large with an adjusted Cohen’s *f* = 0.476 > 0.4.

The results in Table 6 indicate that both groups showed significant improvements from pre-test to post-test. However, the experimental group’s progress was highly significant, whereas the control group’s improvement was modest. No significant difference was observed between the groups on the pre-test, but the experimental group outperformed the control group on the post-test, indicating that the interactive, robot-assisted instruction was more effective than video-based instruction.

### 4.2. Learning Motivation Analysis

To examine the impact of instructional activities on the students’ learning motivation, an independent-samples *t*-test (Table 7) was conducted using a six-item scale measuring intrinsic and extrinsic motivation. Results showed that the experimental group’s mean score was higher than the control group, indicating that the teaching module effectively enhanced the students’ learning motivation with a medium effect size (Cohen’s *d* = 0.51).

To examine differences across dimensional motivation, the scale was separated into intrinsic and extrinsic subscales, and independent-samples *t*-tests were conducted individually. As shown in Table 8, the experimental group performed better than the control group in extrinsic motivation. However, the difference did not reach the level of statistical significance, but the result suggests that interactive robotic operation can still increase the students’ task persistence and learning performance.

In contrast, for intrinsic motivation, the experimental group scored significantly higher than the control group, indicating that hands-on activities effectively enhanced the students’ interest, motivation, and willingness to accept challenges, thereby fostering learning persistence and promoting deeper self-directed exploration in programming logic and problem-solving. The findings suggest that integrating App Inventor 2 with robotic-arm operation significantly enhanced intrinsic motivation and demonstrated a positive trend in extrinsic motivation, thereby supporting further investigation into its broader impacts on learning processes, outcomes, creativity, engagement, and long-term academic development.

Further item-level analysis (Table 9) indicated that within the dimension of extrinsic motivation, both groups reported the highest agreement with the item “Getting a good grade in this course is satisfying”. The experimental group obtained a mean score of 3.89 compared to 3.58 in the control group. Although the difference was not significant, the overall subscale score remained slightly higher in the experimental group.

Within the intrinsic motivation dimension, the highest-rated item for the experimental group was “I feel happy when learning in this course” (M = 4.00), which was significantly higher than the control group’s score (M = 3.48). Other items, including “I feel a sense of accomplishment when I successfully complete the course activities” and “In this course, I enjoy challenging materials that allow me to learn new things” also exhibited a favorable trend for the experimental group.

The qualitative evidence further supported the quantitative findings. For example, students in the experimental group expressed enthusiasm, stating: “It was so cool to see the robotic arm move—I really want to know how it works”, and “When the program failed, I just tried again. It was highly satisfying when the program finally executed successfully”. Such reflections illustrated how interactive and challenging learning contexts fostered curiosity and intrinsic motivation. In comparison, some control group students noted: “It would have been more interesting to directly manipulate the robotic arm”, indicating the motivational value of direct experiential learning. Considered as a whole, these findings suggest that integrating physical practice with visual programming not only enhances intrinsic motivation but also provides potential benefits for extrinsic motivation, thereby supporting multi-faceted motivational development.

### 4.3. Cognitive Load Analysis

To investigate the impact of different instructional activities on the students’ cognitive load, this study utilized an eight-item Likert-scale questionnaire covering two dimensions: mental load and mental effort (Table 10), where a higher score indicated greater cognitive resource demand. The results of an independent-samples *t*-test revealed that the experimental group reported significantly lower cognitive load compared to the control group. The result suggests that interactive instruction with a physical robotic arm can reduce the students’ cognitive load in the short-term, offering initial evidence for its educational value and potential for further refinement.

Further analysis (Table 11) revealed that the experimental group reported a significantly lower cognitive load than the control group in both dimensions of cognitive load. For both mental load and mental effort, the experimental group scored lower than the control group, indicating that the interactive teaching module incorporating robotic-arm operation effectively reduces cognitive load and enhances learning efficiency.

Across all eight cognitive load items, the experimental group consistently reported lower scores than the control group (Table 12). The most notable differences emerged in perceived content difficulty and time pressure during tasks. Within the mental effort dimension, the item “I needed to invest considerable mental effort to complete this activity or achieve its learning goals” also revealed a significant difference, with lower mental effort in the experimental group. For some items, the experimental group showed smaller standard deviations, indicating relatively consistent learning experiences; however, the differences were less evident for other items. This suggests that while the teaching module effectively reduced overall cognitive load, there may still be a certain degree of individual variation among students.

In summary, the instructional design for learning programming logic skills incorporating the robotic arm demonstrated a clear potential to reduce cognitive load and offer more consistent learning experiences. Future research should further leverage interactive feedback, contextual scaffolding, and adaptive support strategies to alleviate the learners’ cognitive load and improve overall learning outcomes.

### 4.4. System Satisfaction Analysis

The experimental group conducted a system satisfaction survey following the intervention to evaluate their learning experience with the interactive teaching module incorporating robotic-arm operation. The questionnaire assessed three dimensions—Perceived Usefulness, Perceived Ease of Use, and Behavioral Intention—with a five-point Likert scale (Table 13). The overall mean score revealed generally positive feedback on the acceptance of instructional materials. Among the three dimensions, Perceived Usefulness received the highest rating, suggesting that the teaching module effectively enhanced the students’ understanding of computational thinking and programming logic. While items in the “Perceived Ease of Use” dimension were rated positively, some students reported operational difficulties, reflecting variations in technological familiarity and user confidence. Behavioral Intention received the lowest mean score, suggesting limited willingness in using the system without external incentives.

According to interview results, some students reported that the robotic arm was expensive and generally unavailable for learning the course content. Other findings highlighted the importance of setting clear learning goals and incorporating gamified elements to sustain user engagement. Overall, the teaching module demonstrated strong potential for enhancing learning outcomes, engagement, and interactive experiences, while further refinements in interactivity and motivational design are recommended.

### 4.5. Qualitative Results

To supplement the quantitative findings, this study conducted post-intervention interviews with students from both groups to explore their perspectives on learning motivation, encountered challenges and problem-solving approaches, evaluations of the instructional materials, and subsequent learning outcomes. Most students indicated that the visualized learning materials, problem-based tasks, and hands-on robotic arm operations effectively stimulated their interest and motivation, while fostering positive attitudes toward learning computational thinking and programming logic skills.

Although some students experienced challenges in grasping spatial concepts and programming logic, they demonstrated adaptability by leveraging peer collaboration and engaging in iterative trial-and-error processes. A few students reported difficulties due to limited classroom visibility and restricted access to equipment for after-class practice, indicating the need to enhance the accessibility and flexibility of hands-on resources. Overall, students provided positive evaluations of the instructional guidance and interface design, stating that the robotic instructional activities effectively enhanced their computational thinking and programming logic skills.

Several students expressed a strong sense of accomplishment and motivation in further learning, such as exploring advanced programming languages or sharing their experiences with others. These findings revealed that the teaching materials successfully fostered learning interest, operational confidence, and sustained motivation. Future instructional designs should focus on enhancing interactivity, providing adaptive support, and expanding opportunities for self-directed, collaborative learning to further improve learning outcomes and promote long-term and self-directed learning.

While most students responded positively to the tasks, a subset reported initial frustration when programming logic or spatial concepts were challenging. Interestingly, this frustration sometimes stimulated creative problem-solving strategies, such as inventing alternative coding sequences or unconventional ways to manipulate the robotic arm, demonstrating that difficulties can unexpectedly catalyze deeper, more persistent, and highly adaptive innovation rather than solely hindering learning. Some students mentioned feeling discouraged when comparing their progress to peers who mastered tasks more quickly. Conversely, this comparison motivated others to seek collaboration and peer guidance, revealing that social dynamics played a divergent role—both challenging and enhancing learning motivation depending on individual coping strategies.

## 5. Discussion

### 5.1. Learning Effectiveness and Conceptual Understanding

The findings reveal that integrating a physical robotic arm with a visual programming environment significantly enhanced the students’ achievement in computational thinking, programming logic, and spatial reasoning. Although both the experimental and control groups showed improvement, the experimental group demonstrated a greater learning gain and smaller score variance, suggesting improved instructional efficiency and reduced learning disparities. These outcomes align with constructivist learning theory [59], which emphasizes that learners actively construct knowledge through the interaction and manipulation of physical tools. The tangible connection between programming commands and robotic operation enabled students to visualize abstract algorithmic processes, transforming symbolic code structures into observable, meaningful actions.

Furthermore, the dual-channel learning framework proposed by Mayer’s Cognitive Theory of Multimedia Learning [60] helps explain why students benefited from the integration of visual and kinesthetic elements. The visual programming blocks in App Inventor 2 reduced the cognitive demands associated with syntax and textual programming, allowing students to focus on logical reasoning and problem-solving strategies. This multimodal engagement—seeing, manipulating, and reflecting—enabled students to process information through both visual and experiential channels, deepening conceptual understanding. The large effect size (η^2^ = 0.360) supports the interpretation that embodied and multimodal learning environments are especially effective for novice programmers, helping them bridge the gap between abstract logic and tangible outcomes.

From an instructional perspective, these findings suggest that incorporating physical interfaces into programming instruction can foster active, situated learning experiences that are accessible and inclusive. By grounding programming logic in concrete operations, students can connect abstract computational concepts to observable outcomes, thereby enhancing their confidence and conceptual understanding.

### 5.2. Motivation and Engagement in Interactive Learning

The motivational results revealed that the experimental group scored significantly higher in intrinsic motivation and showed a positive trend in extrinsic motivation, indicating that hands-on, interactive learning can foster both enjoyment and achievement-oriented drive. According to Self-Determination Theory [38], intrinsic motivation flourishes when learning environments fulfill the needs for autonomy, competence, and relatedness. The robotic programming activities provided learners with opportunities to control the system (autonomy), witness successful task execution (competence), and engage collaboratively or through shared excitement (relatedness).

The qualitative findings reinforce this theoretical interpretation: students expressed enthusiasm and persistence, describing satisfaction when the robotic arm executed their programmed commands successfully. Such reflections highlight the emotional and motivational benefits of embodied learning experiences. As Fredricks et al. [61] noted, affective engagement is a critical driver of cognitive persistence and deeper learning. The hands-on design not only stimulated curiosity but also created a safe environment for trial and error, promoting a growth mindset and resilience.

In contrast, the control group, which relied on lecture-based instruction and video demonstrations, lacked these experiential affordances. Consequently, their engagement was primarily extrinsically driven, focusing on performance outcomes rather than the learning process itself. The difference underscores the pedagogical value of interactive learning environments that balance challenge and autonomy. Such environments not only boost immediate motivation but also foster lasting interest in computational thinking—an outcome essential for sustaining learning trajectories.

### 5.3. Cognitive Load Reduction and Learning Efficiency

The experimental group reported significantly lower mental load and mental effort compared to the control group, indicating that the integration of robotic-arm operation effectively reduced cognitive strain. This finding is well-explained by CLT [62], which distinguishes between intrinsic, extraneous, and germane cognitive load. The physical and visual affordances of robotic-arm operation likely reduced the extraneous load by externalizing abstract processes and providing immediate visual feedback. Rather than mentally simulating the effects of code, students could observe them directly through robotic actions, freeing cognitive resources for meaningful learning.

Moreover, the structured design contributed to higher germane load, as mental effort was directed toward constructing and refining mental schemas rather than coping with unnecessary complexity. The reduction in standard deviation among the experimental group’s scores also suggests that the module provided a more consistent and supportive cognitive environment, benefiting students with varied prior experiences.

These findings contribute to the expanding evidence that interactive and embodied systems can function as cognitive scaffolds in STEM and technology education [63]. By integrating visual programming with real-world interaction, the teaching module balanced cognitive demands, enabling learners to efficiently connect procedural and conceptual knowledge. For educators, this suggests that technological tools should be designed not only to engage students but also to optimize cognition, aligning interface design and feedback mechanisms with human working-memory architecture.

### 5.4. System Satisfaction and Educational Implications

The system satisfaction results indicated generally positive acceptance among students, particularly in perceived usefulness, suggesting strong recognition of the system’s educational value. However, lower scores for perceived ease of use and behavioral intention reflect practical challenges, such as the robotic arm’s cost, availability, and operational complexity. According to Venkatesh and Davis [47], users’ acceptance of educational technology depends on its perceived usefulness and ease of use. The results imply that while the system effectively supports learning, sustained adoption would require the simplification of interfaces, increased accessibility, and clear instructional guidance.

From an educational perspective, this study highlights several key implications. First, integrating physical devices, such as a robotic arm, into programming instruction can make abstract programming logic tangible [1,6], thereby enhancing comprehension and engagement. Second, the combination of hands-on operation and visual feedback can reduce cognitive load and increase inclusivity, especially for beginners or students with weaker abstract reasoning skills. Third, the motivational benefits suggest that embodied learning may serve as a bridge to deeper, self-regulated learning behaviors, ultimately strengthening learners’ long-term engagement with technology education.

In broader terms, this research contributes to the growing recognition that embodied and interactive technologies can support not only technical skill acquisition but also the affective and cognitive dimensions of learning. By situating programming within tangible, feedback-rich contexts, the robotic-arm operation transforms abstract computational reasoning into an engaging, comprehensible, and equitable learning experience [31]. Ultimately, the findings extend beyond statistical significance to emphasize an educational argument: when learning systems are designed to integrate sensory experience, cognitive scaffolding, and motivational support, they empower students to construct knowledge more deeply and persistently [52]. The proposed instructional model thus represents a promising direction for advancing computational literacy and fostering active, motivated, and cognitively efficient learners in the era of intelligent and interactive education.

## 6. Conclusions and Future Work

### 6.1. Conclusions

This study integrated App Inventor 2 with a physical robotic arm to investigate the effects of task-based instructional design on junior high students’ learning outcomes, motivation, and cognitive load. Synthesizing quantitative and qualitative findings, the study yielded several key conclusions that are consistent with prior research in computational thinking, educational robotics, and constructionist learning theories.

(1) Effects on Learning Outcomes

The experimental group demonstrated significantly higher post-test achievement than the control group, indicating that hands-on manipulation of the robotic arm enhanced the comprehension of abstract programming logic and spatial reasoning. These results corroborate Papert’s constructionism framework, which posits that learning is deepened when learners actively construct and manipulate tangible artifacts [5]. Consistent with prior findings on robotics-based instruction [31,34], students developed stronger computational thinking, problem-solving skills, and the ability to translate logical commands into three-dimensional spatial actions, emphasizing the value of embodied learning in programming education.

(2) Effects on Learning Motivation

The experimental group exhibited higher intrinsic motivation, autonomy, and engagement, reflecting the principles of Self-Determination Theory [38]. Qualitative data revealed that immediate visual and kinesthetic feedback from the robotic arm reinforced competence and task ownership, echoing prior research on the motivational impact of gamified and interactive programming activities [21]. Students valued the sense of achievement gained through iterative problem-solving, indicating that hands-on robotics fosters sustained engagement beyond passive learning modalities [35,36].

(3) Effects on Cognitive Load

The experimental group reported lower levels of mental load and mental effort compared with the control group. This finding suggests that the interactive operation of the robotic arm, combined with real-time feedback, effectively alleviated the cognitive burden associated with abstract logic concepts. Dynamic visual feedback from the robotic arm enabled students to understand programming logic more intuitively, minimizing trial-and-error and cognitive load, and thereby enhancing overall learning efficiency.

The experimental group reported lower mental load and effort compared with the control group, suggesting that concrete, manipulable learning tools can reduce extraneous cognitive load and facilitate schema acquisition, as theorized in Cognitive Load Theory [41,42,43]. Real-time visual feedback from the robotic arm enabled students to detect errors immediately, reducing cognitive strain associated with abstract logic manipulation, in line with multimedia learning principles [60]. This supports the notion that integrating physical artifacts with programming tasks can make abstract concepts more concrete, enhancing learning efficiency and knowledge retention.

(4) Student Feedback

Semi-structured interviews highlighted the experiential and affective dimensions of learning, revealing that students perceived the tasks as enjoyable, meaningful, and directly relevant to computational problem-solving. These insights align with constructionist perspectives and prior studies emphasizing active engagement and iterative design in programming education [2,53]. However, some students reported operational challenges and equipment constraints, indicating that effective scaffolding, adaptive guidance, and accessible resources remain critical for equitable outcomes.

(5) Limitations and Implications

The sample consisted of eighth-grade students from a junior high school in Hsinchu, Taiwan, with relatively homogeneous backgrounds, which restricts the external validity and limits the ability to generalize findings beyond similar contexts [25,26,27]. This highlights a core limitation in the study’s design, as the intervention’s effects may be contingent on specific cultural, educational, or cognitive characteristics of the participants. To address this, future research should recruit students from multiple schools and regions, ensuring diverse socioeconomic, cultural, and prior-experience backgrounds, and stratify participants based on baseline skills such as spatial reasoning or programming ability.

The instructional intervention lasted only three weeks, focusing primarily on basic concepts and task operations, without evaluating higher-order skill development or long-term learning outcomes. This short duration constrains the theoretical claims regarding the module’s capacity to enhance deep computational thinking or interdisciplinary integration. Future studies could implement longer interventions with scaffolded, progressively complex tasks and include follow-up assessments to measure retention, transfer, and the sustainability of learning gains.

In the experimental group, the students’ interactions with the robotic arm may have been affected by equipment proficiency, interface adaptation, and operational challenges, which could have introduced variability in implementation fidelity and measurement accuracy. To mitigate these issues, future procedures could include structured training sessions, standardized operation guides, pre-intervention practice opportunities, and logging of task completion times and error rates to objectively verify performance.

Variations in prior programming experience, robotics exposure, or spatial reasoning may have contributed to observed differences in responses and short-term gains, suggesting the need for stratified designs and covariate controls in future studies [28]. Incorporating pre-assessment stratification, covariate controls in statistical analyses, and triangulation with behavioral observations and performance data would enhance methodological rigor and strengthen theoretical interpretations. By explicitly acknowledging these limitations and proposing concrete procedures to address them, future research can improve both the reliability and generalizability of the findings.

### 6.2. Future Work

This study integrated App Inventor 2 with a physical robotic arm to develop a teaching module, and the preliminary results indicate that it effectively fostered junior high students’ computational thinking and programming logic skills while enhancing learning motivation and reducing cognitive load. Overall, the students provided positive evaluations of both the instructional design and the teaching module’s usefulness. However, this study was limited by constraints such as instructional time, the depth and scope of learning materials, and characteristics of participant groups. To promote further academic understanding, future research could be refined and extended in the following directions.

(1) Enhancing Learning Content

At present, the learning content primarily focuses on basic programming skills and spatial concepts. Future development could expand to advanced logic structures, such as variables and nested loops, as well as mathematical modeling and real-life applications. In addition, incorporating gamification elements and more intuitive user interfaces could further enhance interactivity and student motivation.

(2) Extending Instructional Duration

The current course spanned only three weeks with a total of 150 min, allowing for the observation of short-term effects but limiting insights into long-term learning outcomes and the development of deeper skills. We recommend extending the instructional duration, implementing progressively staged and increasingly challenging tasks to reinforce skill mastery, and incorporating delayed post-tests or follow-up surveys to more comprehensively capture the retention and transfer of learning outcomes over time.

(3) Personalized Instruction

Future studies should consider subgroup analyses based on factors such as pre-test scores and individual learning styles to examine the differential effects of the teaching module across student ability levels. Developing personalized instructional strategies could further enhance adaptability and promote greater equity in learning experience.

(4) Cross-Disciplinary Applications

The proposed instructional module demonstrates considerable potential for integration with science, technology, engineering, and mathematics domains, including geometric angle control, physical force simulation, and motion trajectory prediction. These applications can enhance students’ comprehension of abstract concepts while enhancing their abilities in cross-disciplinary integration. Future research could focus on designing and evaluating interdisciplinary learning scenarios to enhance educational outcomes while amplifying societal and interdisciplinary impacts. In addition, it is important to present the results for boys and girls to analyze potential differences in learning outcomes by sex.

Future development should focus on extending the instructional time, deepening the learning content, optimizing the user interface, and broadening the scope of applications while addressing learner diversity and monitoring long-term outcomes. This approach can enable technology-enhanced instruction to integrate innovation with practicality, thereby delivering greater educational value across diverse learning contexts.

## Figures and Tables

**Table 1:** Two-way specification table for the achievement test.

Learning Topics	Cognitive Level	Test Items	Supporting References
Robots and Spatial Coordinates	Understanding	2, 3, 5	[1,7,31,33,34]
	Applying	1, 4	[1,7,31]
	Analyzing	—	[1,31,34]
Computational Thinking	Understanding	8	[6,19,20,53]
	Applying	6, 10	[6,20,53,55]
	Analyzing	7, 9	[6,20,53]
Programming Logic	Understanding	13	[9,10,14]
	Applying	11, 12, 14, 15	[9,10,14,15]
	Analyzing	—	[9,14,15]

**Table 2:** Procedure for the quasi-experimental study.

Group	Pre-Test	Treatment	Post-Test
Experimental Group	O1	X1	O2, O3, O4
Control Group	O1	X2	O2, O3

**Table 3:** Descriptive statistics of pre- and post-test scores for the two groups.

Group		Pre-Test		Post-Test	
		Mean	SD	Mean	SD
Experimental	Understanding	2.95	1.03	3.54	0.77
Control	Understanding	2.59	1.21	2.95	0.94
Experimental	Applying	4.00	1.58	5.35	1.03
Control	Applying	3.43	1.48	3.76	1.40
Experimental	Analyzing	0.51	0.61	0.84	0.55
Control	Analyzing	0.59	069	0.59	0.55
Experimental	Total Score	7.45	2.29	9.73	1.50
Control	Total Score	6.62	1.97	7.29	1.83

**Table 4:** Paired-samples *t*-test results of learning achievement tests for the two groups.

Group	Cognitive Level	Gain	SD	SE	*t*	*p*
Experimental	Understanding	0.59	0.77	0.13	4.65	<0.001 ***
Control	Understanding	0.36	0.94	0.16	2.32	0.026 *
Experimental	Applying	1.35	1.03	0.17	7.99	<0.001 ***
Control	Applying	0.33	1.40	0.23	1.43	0.161
Experimental	Analyzing	0.33	0.55	0.09	3.67	<0.001 ***
Control	Analyzing	0.00	0.55	0.09	0.00	1.00
Experimental	Total Score	2.27	1.50	0.25	9.27	<0.001 ***
Control	Total Score	0.67	1.83	0.30	2.23	0.032 *

* *p* < 0.05, *** *p* <0.001.

**Table 5:** Results of two-factor analysis of variance for mixed designs.

Source	Type III Sum of Squares	df	F	*p*	η^2^
Test	80.277	1	40.503	0.000 ***	0.360
Group	98.926	1	18.253	0.000 ***	0.202
Test × Group	23.520	1	11.867	0.001 **	0.141
Between Subjects	390.216	72			
Residuals	142.703	72			
Sum	735.642	147			

** *p* < 0.01, *** *p* < 0.001.

**Table 6:** Summary of simple main effects from the mixed design.

Primary Effect	Type III Sum of Squares	df	F	*p*
Experimental Group	95.351	1	37.454	0.000 ***
Control Group	8.446	1	5.956	0.020 *
Error	142.703	72		
Pre-test	12.986	1	2.834	0.097
Post-test	109.459	1	38.818	0.000 ***
Error	390.216	72		

* *p* < 0.05, *** *p* < 0.001.

**Table 7:** Independent-samples *t*-test results on learning motivation between the two groups.

Group	Mean	SD	df	*t*	*p*
Experimental Group (*n* = 37)	3.71	0.56	72	2.206	0.03 *
Control Group (*n* = 37)	3.41	0.61			

* *p* < 0.05.

**Table 8:** Independent-samples *t*-test results on extrinsic and intrinsic motivation between groups.

Group	Extrinsic Motivation		*p*	Intrinsic Motivation		*p*
	Mean	SD		Mean	SD	
Experimental Group	3.66	0.62	0.075	3.77	0.64	0.031 *
Control Group	3.40	0.62		3.43	0.69	

* *p* < 0.05.

**Table 9:** Statistical results of individual items in learning motivation for the two groups.

Dimension	Question	Experimental Group		Control Group		*p*
		Mean	SD	Mean	SD	
Extrinsic Motivation	Getting a good grade in this course is satisfying.	3.89	0.698	3.58	0.818	0.088
	2. If possible, I would like to get a higher grade than other students in this course.	3.62	0.794	3.41	0.818	0.257
	3. I hope to perform well in the course so that I can demonstrate my abilities in front of others.	3.45	0.691	3.45	0.691	0.153
Overall extrinsic motivation: mean and standard deviation		3.66	0.64	3.40	0.62	0.075
Intrinsic motivation	4. I feel happy when learning this course.	4.00	0.707	3.48	0.789	0.004 **
	5. I feel a sense of accomplishment when I successfully complete the course activities.	3.67	0.818	3.46	0.789	0.249
	6. In this course, I enjoy challenging materials that allow me to learn new things.	3.64	0.823	3.35	0.777	0.119
Overall intrinsic motivation: mean and standard deviation		3.77	0.64	3.43	0.69	0.031 *
Overall learning motivation: mean and standard deviation		3.71	0.56	3.41	0.61	0.030 *

* *p* < 0.05, ** *p* < 0.01.

**Table 10:** Independent-samples *t*-test results on overall cognitive load for both groups.

Group	Mean	SD	*t*	*p*	Cohen’s *d*
Experimental Group (*n* = 37)	2.60	0.58	−3.401	0.001 **	0.78
Control Group (*n* = 37)	3.02	0.50			

** *p* < 0.01.

**Table 11:** Independent-samples *t*-test results on mental load and mental effort within groups.

Group	Mental Load		*p*	Mental Effort		*p*
	Mean	SD		Mean	SD	
Experimental Group	2.65	0.65	0.002 **	2.51	0.67	0.004 **
Control Group	3.07	0.51		2.94	0.61	

**p < 0.01.

**Table 12:** Statistical results of individual items in the cognitive load questionnaire for both groups.

Dimension	Question	Experimental Group		Control Group		*p*
		Mean	SD	Mean	SD	
Mental Load	The learning content in this activity was difficult for me.	3.10	0.90	3.76	0.70	0.001 **
	2. I had to put a lot of mental effort into answering the questions in this learning activity.	2.94	0.81	3.23	0.70	0.107
	3. I felt confused when learning the content of this activity.	2.59	0.95	3.00	0.85	0.055
	4. Solving problems in this activity were frustrating to me.	2.37	1.00	2.56	0.78	0.376
	5. There was not enough time for me to complete the questions in this activity.	2.24	0.79	2.82	0.75	0.002 **
Overall mental load: mean and standard deviation		2.65	0.65	3.07	0.51	0.002 **
Mental Effort	6. The teaching method or materials in this activity were difficult for me to follow.	2.59	0.79	2.94	0.79	0.056
	7. I needed to invest considerable mental effort to complete this activity or reach its learning goals.	2.48	0.76	3.17	0.85	0.000 ***
	8. I found the teaching method in this learning activity difficult to understand or follow.	2.45	0.80	2.71	0.79	0.162
Overall mental effort: mean and standard deviation		2.51	0.67	2.94	0.61	0.004 **
Overall cognitive load: mean and standard deviation		2.60	0.58	3.02	0.50	0.001 **

** *p* < 0.01, *** *p* < 0.001.

**Table 13:** System satisfaction on individual questionnaire items.

Dimension	Question	Mean	SD
Perceived Usefulness	I believe instructional materials clarify the principles of robotics.	4.08	0.587
	2. I believe the instructional materials assist in comprehending the concepts of programming logic.	4.03	0.677
	3. Learning through instructional materials is beneficial for acquiring new knowledge.	3.87	0.578
Overall Perceived Usefulness: Mean and Standard Deviation		3.99	0.614
Perceived Ease of Use	4. I can easily use the instructional materials to complete the tasks in the course.	3.43	0.948
	5. I find the interface of the instructional materials easy to use.	3.55	0.724
	6. Overall, the instructional materials used in this learning activity are helpful for learning and easy to use.	3.74	0.724
Overall Perceived Ease of Use: Mean and Standard Deviation		3.57	0.799
Behavioral Intention	7. I am willing to use this instructional material when I need to learn related content in the future.	3.42	0.722
	8. I am willing to learn using this teaching method and instructional material.	3.39	0.823
	9. I would actively use this instructional material to learn the course content.	3.13	0.777
Overall Behavioral Intention: Mean and Standard Deviation		3.31	0.774
Overall System Satisfaction: Mean and Standard Deviation		3.63	0.73

## Data Availability

Data are available on request due to restrictions.

## Appendix A. Achievement Test

**Part A. Robot and Spatial** **Coordinates**

(1) If James wants the robotic arm to draw a straight line on the table, how should he operate the robotic arm?
  A. Only rotate Joint 1
  B. Only rotate Joint 2
  C. Only rotate Joint 3
  D. Rotate both Joint 2 and Joint 3
(2) David designed a Snake game path in App Inventor 2, aiming for it to follow the alphabetical order A→B→C…, as shown in the following figure. If the snake starts from A(2,10), what will be the coordinates of the first point where it touches the X-axis?
  A. (8, 0)
  B. (12, 0)
  C. (16, 0)
  D. (20, 0)
(3) If the robotic arm is to pick up the blue object on the table as shown below, which type of rotational motion is required for the three joints?
  A. Joint 1 clockwise, Joint 2 clockwise, and Joint 3 counterclockwise
  B. Joint 1 clockwise, Joint 2 counterclockwise, and Joint 3 clockwise
  C. Joint 1 counterclockwise, Joint 2 clockwise, and Joint 3 counterclockwise
  D. Joint 1 counterclockwise, Joint 2 clockwise, and Joint 3 clockwise
(4) To move the robotic arm’s end effector from the yellow grid to the blue grid, which joint will not be used?
  A. Joint 1
  B. Joint 2
  C. Joint 3
  D. Joint 4
(5) A robot’s base is positioned at the origin (0, 0, 0), and its end effector is initially located at coordinates (0, 5, 2). When Joint 1 rotates 90° clockwise, the arm is raised by 2 units and extended forward by 3 units. What will be the new coordinates of the end effector?
  A. (8, 2, 5)
  B. (8, 0, 4)
  C. (5, 8, 5)
  D. (8, 5, 5)

**Part B. Computational** **Thinking**

(1) A robot begins at position (0, 0) on a two-dimensional plane, facing right along the positive X-axis. Using block-based programming to control its movement, if the following commands are executed in sequence, what will be its final position and orientation?
  - Turn right 90°
  - Move forward 10 units
  - Turn left 90°
  - Move forward 5 units
  What is the final x-coordinate of the robot?
  A. 15
  B. 10
  C. 0
  D. 5
(2) Tom used his program to control four robots moving within the grid shown in the figure below, where A, B, C, and D represent charging stations. The program may include the following three commands:
  - Step forward—move to the next grid in front
  - Turn left—rotate 90° counterclockwise, but do not move forward
  - Turn right—rotate 90° clockwise, but do not move forward

Tom wrote the program: “Step forward, turn left, step forward, step forward” and downloaded it to all four robots. To ensure that each robot reaches a charging station without collisions, Tom must carefully select appropriate starting positions and set their initial orientations before running the program. Which grid location should Tom avoid placing a robot in? Note: Once the program starts, all robots execute their commands simultaneously.

A. Grid 2
B. Grid 3
C. Grid 4
D. Grid 5

(3) George learned the following four drawing commands and a control command in his computer graphics class:
  - T5—draw a large triangle
  - T1—draw a small triangle
  - R—draw a square
  - Z—draw a trapezoid
  - [ ]#—repeat the commands inside [ ] # times
  George drew the following pattern. Which sequence of commands did he use?
  A. [T5, R, T1, T1]2 R
  B. T5, Z [T1]2, Z, [T1]2, R
  C. [T5, Z, T1]2 T1, T1, R
  D. [T5, Z, [T1]2]2, R
(4) A robot vacuum is exploring an unknown environment that may contain obstacles. Equipped with sensors (e.g., ultrasonic sensors), it can detect both the distance and direction of obstacles. Using block-based programming to control its movement, which of the following strategies would be most effective for enabling the robot to navigate and explore the environment efficiently?
  A. Randomly select a direction and move, repeating many times.
  B. Move following a fixed pattern (e.g., square or spiral) until all areas are explored.
  C. Keep moving forward until encountering an obstacle, then randomly choose a direction to bypass it, repeating the process.
  D. Combine sensor data with pre-set map information to plan an optimal path.
(5) The control commands of a robot vacuum are defined as follows:
  - Move Forward *n*: The robot moves forward *n* grids (*n* is a positive integer).
  - Move Right: The robot first turns right, then moves forward one grid.
  - Move Left: The robot first turns left, then moves forward one grid.
  - Repeat *R*: The robot repeats commands inside the brackets *R* times (*R* is a positive integer).

After the robot vacuum passed over a heavily soiled floor, it left a visible track marking the cleaned grids. Roger programmed the robot with the following instructions: Repeat 2 (Move Forward 2, Move Right), Move Forward 2, Move Right, Repeat 2 (Move Forward 1), Move Right. Which of the figures (A, B, C, or D) correctly illustrates the path traced by the robot after executing the above instructions?

**Part C. Programming** 

(1) When a robotic arm performs a task, it evaluates sensor readings to determine its actions. Suppose there is a color sensor variable and a shape sensor variable, each storing text representing the detected color or shape of an object. Which of the following programs correctly implements the behavior: if the color sensor reads “Red” and the shape sensor reads “Square”, the label displays “This is a red square”; otherwise, it displays “This is red”?

(2) In the maze shown below, the robot arm’s end position, indicated by the yellow block, moves from the position in the left figure to the position in the right figure. Which App Inventor 2 program correctly moves the robotic arm’s end position to the target location?

(3) What value is produced by the following program?
  A. Common multiple
  B. Least common multiple (LCM)
  C. Common factor
  D. Greatest common factor (GCF)

(4) Which program can replace the one shown below while maintaining the same functionality?

(5) The maze below was created in App Inventor 2 using a list. Red and green squares indicate obstacles, and the yellow square shows the robot’s current position. The variable lastMove is set to “Up.” After executing the following program, which of the figures (A, B, C, or D) represents the updated maze state?
